# The relationship between serum vitamin D level and premenstrual syndrome in Iranian women

**Published:** 2016-10

**Authors:** Samira Rajaei, Azadeh Akbari Sene, Sara Norouzi, Yasrin Berangi, Sahereh Arabian, Parvaneh Lak, Ali Dabbagh

**Affiliations:** 1 *School of Medicine, Tehran University of Medical Sciences, Tehran, Iran. *; 2 *Department of Obstetrics and Gynecology, Shahid Akbar-abadi Hospital IVF Center, Iran University of Medical Sciences, Tehran, Iran.*; 3 *Anesthesiology Research Center, Shahid Beheshti University of Medical Sciences, Tehran, Iran. *

**Keywords:** *Vitamin D*, *Premenstrual syndrome*, *Body mass index*, *Menarche*, *Premenstrual dysphonic disorder*

## Abstract

**Background:**

Premenstrual syndrome (PMS) is among the most unfavorable problems in women in reproductive age; however its pathophysiology is still not fully confirmed. Vitamin D as an immunomodulator could prevent inflammatory state before and during menstruation.

**Objective:**

The aim was to investigate whether there is any relationship between serum vitamin D levels and PMS.

**Materials and Methods:**

In total, 82 women participate in this case-control study which was conducted in Shahid Akbar-abadi hospital from November 2013 to March 2015. Categorization was based on an Iranian version of the premenstrual symptoms screening tool (PSST). Levels of 25 hydroxy-vitamin D3 (25OHD) were determined by using 25-OH Vitamin D ELISA kit in luteal phase. Characteristics of participants and vitamin D levels were compared between two groups by using independent sample t-test.

**Results:**

Menarche age of women with PMS was significantly lower than normal women (p=0.04). Body mass index was not statistically different between groups. We observed a high rate of vitamin D deficiency and also its severe deficiency in both PMS and non-PMS groups. However, our study demonstrated no significant difference in the levels of serum 25OHD between the two groups.

**Conclusion:**

It seems there is no association between PMS and serum levels of vitamin D3; however, the high rate of vitamin D deficiency among young Iranian women emerges special health care considerations in this group.

## Introduction

Premenstrual syndrome (PMS) is among the most unfavorable problems in women with reproductive age; which imposes a great deal of economic burden to the society. Although many hypotheses have been proposed for its pathophysiology, none are still fully confirmed. The effects of progesterone and Gamma Amino Butyric Acid (GABA) in the symptom creation have been established; however, definitive treatment for this syndrome is not well known up to now (1).

One of the hormones which were proposed to have a preventive role in PMS is vitamin D3 (2). Previous data support the idea that vitamin D3 could lower the risk of PMS; however, the mechanisms which underlie these desirable effects are not fully understood (3). Vitamin D as an immunomodulator could prevent inflammatory state before and during menstruation and also could alter neurotransmitters’ function (4, 5).

 This study was performed to assess the possibility of relationship between PMS and serum levels of 25 hydroxy-vitamin D3 (25OHD) in luteal phase of young women in reproductive age, during the zenith of irritating symptoms.

## Materials and methods

In this cross sectional, case-control study, 82 women were selected among the 18-45 years old women who referred for routine gynecologic investigation to a gynecologic clinic from November 2013 to March 2015 in a university affiliated hospital, Tehran, Iran. The study was approved by the ethics institutional review board of Tehran University of Medical Sciences. The study was granted by Research Deputy, Tehran University of Medical Sciences. . The aim and procedure of the study was described for these referrers by a gynecologist. Women who signed informed consent participated in the study. 

Diagnosis of premenstrual syndrome was based on an Iranian version of premenstrual symptoms screening tool (PSST) (6). According to this tool we designed a questionnaire and used it for diagnosis of PMS. A part of consisted questionnaire was related to general health and gynecologic status of the participants. All participants completed the questionnaire and according to their responses, they were categorized to one of two groups; PMS or control. As the number of premenstrual dysphoric disorder (PMDD) cases was low, they were categorized in PMS group.

Exclusion criteria were any contained endocrine disorders (thyroid dysfunction, polycystic ovary syndrome, menstrual cycle disturbances, diabetes mellitus), history of previous documented gynecologic disorders, history of previous documented autoimmune diseases, and any consumption of these medications: oral contraceptive agents, vitamin D supplements, corticosteroids and anti-depressant drugs within 6 months of the study.

Five mm blood samples were taken from each person in the luteal phase according to their reported last menstrual period. Serums were separated and stored at -20°C until all samples were collected. Levels of 25OHD were determined by using 25-OH Vitamin D ELISA kit (Euroimmun, Luebeck, Germany) according to manufacturer’s instructions. The reference ranges of plasma 25OHD were mentioned as follows: very severe vitamin D deficiency; <5 ng/ml, severe vitamin D deficiency; 5-10 ng/ml, vitamin D deficiency; 10-20 ng/ml, suboptimal vitamin D provision; 20-30 ng/ml, optimal vitamin D level; 30-50 ng/ml, upper normal; 50-70 ng/ml, overdose but not toxic; 70-150 ng/ml and vitamin D intoxication; as >150 ng/ml.

**Statistical analysis**

For data analysis, SPSS software Version 16 was used (SPSS, Inc. Chicago, IL, USA). After confirming the normality of data distribution with Kolmogorov-Smirnov test, we use independent sample t-test for comparing the levels of 25OH vit D3 between two groups. P<0.05 were considered statistically significant. For drawing the figures, GraphPad software Version 5 was used (GraphPad software inc. La Jolla, CA, USA).

## Results

Eighty two women participated in this study and completed the questionnaire. Nine cases were excluded from the study according to the exclusion criteria. Basic variables of the two study groups are demonstrated in table I.

The results demonstrate that the majority of PMS patients (87.5%) and normal women (84.4%) were vitamin D deficient. Vitamin D levels in the PMS group were as follows: 17.1% very severe deficiency, 39.0% severe deficiency, 31.7% deficiency, 2.4% suboptimal, 4.9% optimal and 4.9% with upper normal levels. In the control (non-PMS) group the levels were as follows: 31.1% very severe deficiency, 28.1% severe deficiency, 25.0% deficiency, 3.1% suboptimal, 9.4% optimal and 3.1% with upper normal levels.

**Figure 1:**
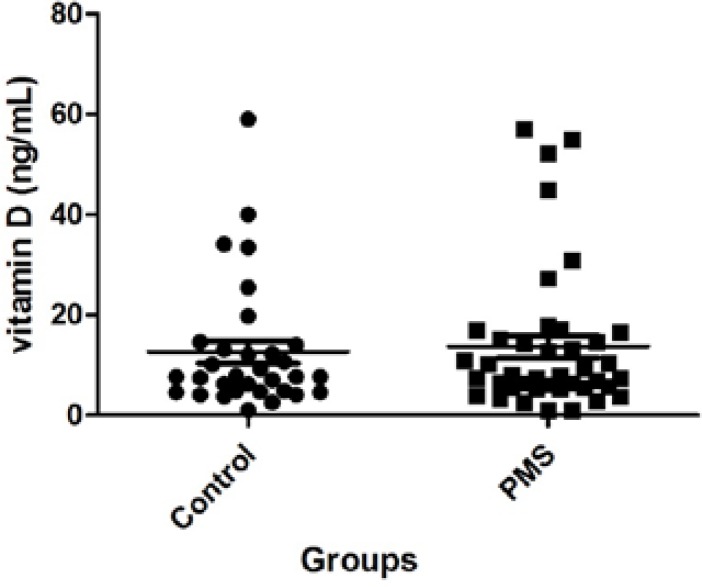
Serum levels of Vitamin D in the two study groups

The difference of mean serum 25OHD level was not statistically significant between groups as shown in figure 1 (13.74±2.24 in PMS vs. 12.67±2.25 ng/mL in control). Also there was no significant difference in the severity of 25OHD deficiency between groups. We also found no meaningful relationship between the severity of vitamin D level deficiency and the severity of PMS symptoms.

**Table I:** Basic variables of the two study groups of participants

**Groups**	**PMS**	**Control**	**p-value**
Number	41	32	-
Age (years)	28.46 ± 4.31	31.25 **±** 7.39	0.64
Menarche age (years)	12.65 ± 1.46	13.37 ± 1.47	0.04
Body mass index	22.72 ± 2.89	23.93 ± 2.57	0.12

Data presented as (Mean ± SD)

PMS: Premenstrual syndrome

## Discussion

Basic variables’ findings indicated that only menarche age is statistically different between PMS and normal women; however, our study demonstrated no different levels of 25OHD between groups. Study results showed that menarche age of women with PMS is significantly lower than normal women. Unlike this finding, Bertone-Johnson *et al* and Thys- Jacob *et al* showed that there is no difference in menarche age between premenstrual syndrome or premenstrual dysphoric disease and normal women (3, 7). Lower menarche age is associated with many health problems, which could involve premenstrual syndrome (8).

Our findings suggest that body mass index is not statistically different between two groups. Although some studies show a direct linear relation between two parameters, there are studies that do not show this relationship (3, 7, 9, 10). This investigation demonstrated that the levels of 25OHD were not different between PMS patients compared to control group. In concordance with our results Obeidat *et al* indicated that there is not any association between 25OHD levels and premenstrual symptoms among Jordanian females (11). Similarly Bertone-Johnson *et al *in their prospective cohort study explained that 25OHD levels were not related to the risk of PMS and higher vitamin D levels could improve some symptoms related to PMS, but not all of them (12).

Our study could not show any association between 25OHD level in luteal phase and persistence of PMS. One possible explanation for this could be the high prevalence of vitamin D deficiency among both our case and control groups. Several studies showed the high prevalence of vitamin D deficiency in different parts of Iran (13-15). We studied the young women in the city of Tehran. Heshmat and colleagues studied the prevalence of vitamin D deficiency in five major cities of Iran and demonstrated that the prevalence of this deficiency is more evident in Tehran, the capital city of Iran (16). Vitamin D deficiency is very common in Iran and other countries in Middle East and this could be partly related to limited sun exposure due to cultural practices, poor vitamin D supplementation and calcium deficiency in this region (17).

As the main limitation of this study, the high prevalence of vitamin D deficiency in Iran and many other countries in Middle East makes it hard to find a significant relationship between 25OHD deficiency and other pathologies such as PMS and PMDD. Another concept is that there is still no universal agreement about normal levels of vitamin D and its deficiency states. Moradzadeh and colleagues in their study of 5329 blood samples from different areas of Iran, calculated different cut-off values for definition of vitamin D deficiency states among Iranian population (18). However, they also found unexpectedly high prevalence of all stages of vitamin D deficiency among both Iranian women (75.1%) and men (72.1%). Vitamin D is a secosteroid hormone with known calcium regulatory effects. Also, it shows anti- proliferative, pro-differentiative and immunomodulatory properties (19). Although the main source of vitamin D is skin biosynthesis, a part of which is supported by diet. Vitamin D deficiency is prevalent worldwide and it includes Iranian reproductive age women (20).

Although our study could not show different levels of vitamin D between PMS and normal women, since PMS is associated with increased inflammatory cytokines, anti- inflammatory metabolites such as vitamin D could be able to improve some symptoms of PMS (4). The high rate of vitamin D deficiency among young Iranian women emerges special health care considerations in this group.
